# Comparative Metabolomic Profiling of Resistant and Susceptible *Coffea arabica* Accessions to Bacterial Pathogen Infection

**DOI:** 10.3390/plants15020216

**Published:** 2026-01-09

**Authors:** Salim Makni, Adrian Heckart, Jean-Christophe Cocuron, Lucas Mateus Rivero Rodrigues, Suzete Aparecida Lanza Destéfano, Masako Toma Braghini, Oliveiro Guerreiro Filho, Ana Paula Alonso

**Affiliations:** 1 Department of Biological Sciences, BioDiscovery Institute, University of North Texas, Denton, TX 76205, USA; salim.makni@unt.edu (S.M.); adrianheckart@my.unt.edu (A.H.); 2 BioAnalytical Facility, University of North Texas, Denton, TX 76205, USA; jeanchristophe.cocuron@unt.edu; 3 Centro de Café Alcides Carvalho, Instituto Agronômico de Campinas, Campinas 13075-630, SP, Brazil; lucasmriverorodrigues@gmail.com (L.M.R.R.); mako.braghini@gmail.com (M.T.B.); oliveiro.guerreiro@sp.gov.br (O.G.F.); 4 Laboratório de Bacteriologia Vegetal, Instituto Biológico, Campinas 13012-970, SP, Brazil; suzete.destefano@sp.gov.br

**Keywords:** metabolites, LC-MS/MS, untargeted metabolomic, *Coffea*, *Pseudomonas coronafaciens* pv. *garcae*, infection, resistance

## Abstract

*Coffea*, a plant species of significant agricultural value used in coffee production, is a key commodity that supports the livelihoods of millions of people worldwide. However, coffee cultivation faces substantial threats from various pathogens, including *Pseudomonas coronafaciens* pv. *garcae* (*Pcg*), the causative agent of bacterial blight. This pathogen compromises coffee plant health, leading to reduced yields and plant death and impacting farmers and large-scale producers. Understanding the mechanisms underlying resistance to *Pcg* in the leaves of the resistant IAC 2211-6 *Coffea arabica* accession is crucial for developing effective control strategies. This study aimed to identify candidate biomarkers of resistance by comparing the leaf metabolome of (i) the resistant IAC 2211-6 and the susceptible IAC 125 RN *Coffea arabica* accessions and (ii) *Pcg*-infected and uninfected leaves. Untargeted metabolomics revealed distinct metabolic profiles between accessions. Flavonoids were more abundant in susceptible leaves. In contrast, resistant leaves showed increased levels of pipecolic acid ethyl ester, a structural derivative of a key systemic acquired resistance signal, and spiropreussione B, a compound associated with fungal endophytes. These findings highlight candidates potentially linked to resistance and suggest that systemic signaling and beneficial microbial interactions may contribute to resilience.

## 1. Introduction

Considered the most widely consumed beverage, coffee is derived from a large diversity of plant species, including *Coffea canephora* and *C. arabica* [1]. These coffee plant species are mainly cultivated in Asia, South America, and East Africa; the higher altitude and cooler temperature, combined with the fertile soil of these regions, promote their optimal growth and development [2]. Among these countries, Brazil is the first and largest producer and exporter of coffee beans worldwide (OEC, 2022). Unlike other *Coffea* species, *C. arabica* holds significant economic importance for three main reasons: (i) Although *C. canephora* (Robusta) and *C. arabica* (Arabica) are the two most prevalent coffee species in Brazil, the latter is cultivated over an area estimated at 1,753,100 hectares, accounting for 60 to 70% of the global coffee production [3]. (ii) The natural recombination between *C. eugenioides* and *C. canephora* has enabled the generation of a broad genetic diversity within the *C. arabica* species [4], resulting in the development of a large variety of *C. arabica* plants [2]. (iii) Arabica coffee exhibits higher nutritional and flavor quality compared to coffee derived from other *Coffea* species. Indeed, *C. arabica* beans are characterized by a higher sugar content and lower concentrations of caffeine and chlorogenic acid compared to *C. canephora* (Robusta). Specifically, Arabica contains approximately 1.5 g of caffeine and 6.8 g of chlorogenic acid per 100 g of coffee, whereas Robusta contains 2.4 g and 8.5 g, respectively. These compounds contribute differentially to coffee bitterness, with caffeine accounting for ~10% and chlorogenic acids for 60–70% of the perceived bitterness [5,6,7]. In addition, Arabica beans are characterized by a higher amount of trigonelline (1.4 g/100 g in Arabica vs. 1 g/100 g in Robusta) [6]. This alkaloid confers various health benefits to humans, such as reducing cardiovascular risks, exhibiting antimicrobial properties, protecting the kidneys and liver from damage, and safeguarding the central nervous system against disorders [8].

*Coffea* species are known to be highly sensitive to climate changes and infection by phytopathogens, which significantly alter their growth, development, and survival [9,10]. Indeed, phytopathogens affect various organs of the coffee plants, increasing the risk of developing diseases. For instance, the “coffee leaf rust” is a disease caused by the fungus *Hemileia vastatrix* [11]. Defoliation triggered by this fungus significantly hampers the coffee plants’ ability to photosynthesize and develop, leading to fewer coffee cherries and, consequently, a lower coffee production yield [11,12]. Considered one of the most significant bacterial phytopathogens affecting *Coffea* plants, *Pseudomonas coronafaciens* pv. *garcae* (*Pcg*) causes the “bacterial halo blight of coffee” [13,14]. *Pcg* infection significantly impacts multiple structures of the coffee tree, including leaves, branches, flowers, and fruits. The bacterium typically invades through natural openings or wounds resulting from pruning, environmental stress, or damage caused by other pathogens. A hallmark of the disease is the formation of water-soaked lesions that initially appear translucent in young plants but later develop into darkened necrotic areas encircled by a characteristic yellowish halo in mature foliage. Over time, these lesions expand, compromising leaf integrity and leading to premature rupture in older leaves. In flowering plants, necrosis may affect the floral rosettes, ultimately triggering defoliation, desiccation of productive branches, and impaired fruit development at early stages. This cascade of pathological effects severely diminishes the plant’s photosynthetic capacity and results in substantial yield losses [14,15].

Despite the overall susceptibility of *Coffea* plants to phytopathogens, a subset of cultivars demonstrates resistance [16,17,18]. A deeper understanding of the mechanisms that confer *Pcg* resistance in the leaves of the resistant IAC 2211-6 *C. arabica* accession is essential for (i) identifying resistance-associated genes, (ii) developing durable and long-term *Coffea* varieties resistant to *Pcg*, (iii) promoting sustainable *Coffea* cultivation in response to evolving pathogens, (iv) enhancing coffee productivity, and (v) minimizing reliance on chemical treatments. This project aimed to (i) detect metabolites highly represented in the leaves of the resistant compared to the susceptible *Coffea* cultivar and (ii) compare the metabolome of *Pcg*-infected leaves with that of uninfected leaves. The objective was to identify bioactive molecules potentially associated with resistance to the infection. Metabolites associated with the plant’s defense are expected to accumulate in the resistant leaves and/or be produced during infection to (i) inhibit *Pcg* growth, (ii) prevent the reduction in photosynthetic capacity caused by bacterial halo blight disease, and (iii) promote the overall plant health. For this purpose, untargeted metabolomics was employed, as this approach enables the detection of trace phytochemicals [19,20,21], as well as the identification of a wide range of metabolites extracted from biological samples [22,23]. Metabolites extracted from plant samples can be analyzed by nuclear magnetic resonance (NMR) and mass spectrometry (MS). The implementation of MS-based metabolomics, particularly the use of a high-resolution mass spectrometer (HRMS) coupled with a high-performance liquid chromatography (HPLC), is preferred due to the higher resolution and sensitivity of this analytical tool, which also allows for the analysis of smaller sample quantities [23,24,25]. Several spectral libraries are also available to facilitate the annotation of compounds detected by HPLC-HRMS [23,26,27]. In addition, untargeted metabolomics of *Coffea* leaves using high-resolution quadrupole–time-of-flight (HR-QTOF) analyzers have previously enabled the detection and annotation of several coffee compounds, such as flavonoids and polyphenols [28]. In the present study, the analysis of the leaf metabolome was performed using an HPLC coupled with an HR-QTOF.

## 2. Results

### 2.1. Pcg Infection Results in Disruptions of Biomass Composition

Biomass composition in coffee leaves was first analyzed in the resistant (R) IAC 2211-6 and susceptible (S) IAC 125 RN leaf accessions, which were either infected with *Pcg* (Pcg IBSBF, infection), infiltrated with sterile distilled water (Mock, control), or untreated to avoid potential wounding from the infiltration process (None, non-infected control). The objective was to assess the impact of *Pcg* infection on key biochemical components, including fatty acids, proteins, cell walls, and starch. In parallel, comparative profiling was conducted across different accessions to determine whether genotypic variation influences biomass composition under both infected and non-infected conditions. No significant change in biomass composition was observed following the mock infiltration of uninfected leaves (Figure S1A–D, “S-None vs. S-Mock” and “R-None vs. R-Mock”), nor between susceptible and resistant leaves (Figure S1A–D, “R-None vs. S-None”, “R-Mock vs. S-None” and “R-Pcg IBSBF vs. S-Pcg IBSBF”). Additionally, no changes were detected in the resistant leaves following *Pcg* infection (Figure S1A–D, “R-None vs. R-Pcg IBSBF” and “R-Mock vs. R-Pcg IBSBF”). However, although fatty acid, protein, and cell wall components were unchanged between S-Mock and S-Pcg IBSBF (Figure S1A,B,D), *Pcg*-infected susceptible leaves exhibited a significant 51% decrease in starch content compared to mock-infiltrated susceptible leaves (Figure S1C, “S-Mock vs. S-Pcg IBSBF”). Furthermore, when comparing infected to non-infiltrated susceptible leaves, a significant 48% increase in protein content was observed, along with a 53% and 32% reduction in starch and cell wall content, respectively (Figure S1B–D, “S-None vs. S-Pcg IBSBF”). Overall, *Pcg* infection significantly altered biomass composition in the susceptible but not in the resistant leaves, likely due to the establishment of defense mechanisms in the resistant accession that prevent pathogen-induced biomass disruption.

### 2.2. Resistance Mechanisms Are Established in the Resistant Coffea Accession Prior to Pcg Infection

An untargeted metabolomic approach was then performed to analyze metabolites extracted from the resistant (R) IAC 2211-6 and susceptible (S) IAC 125 RN leaf accessions under both uninfected and infected conditions to uncover bioactive molecules potentially associated with resistance to *Pcg* infection. Leaf metabolome was therefore analyzed between (i) the resistant and susceptible accessions and (ii) the infected leaves and uninfected controls.

A comprehensive profiling using high-resolution LC-MS/MS-based untargeted metabolomics was performed. Two orthogonal chromatography types—reverse phase (RP) and hydrophilic interaction liquid chromatography (HILIC)—were employed to facilitate the annotation of semi-polar and polar metabolites, respectively, and maximize coverage of the *Coffea* metabolome. A comparative analysis of the *Coffea* leaf metabolome was initially conducted without consideration of feature annotation. LC-MS/MS using sequential window acquisition of all theoretical mass spectra (SWATH-MS), followed by data processing in MS-DIAL 5, enabled the detection of 14,021 features in HILIC negative mode, 12,169 in HILIC positive mode, 8552 in RP negative mode, and 10,486 in RP positive mode. Subsequent filtering using the MS-CleanR module of MS-DIAL 5 reduced the number of detected features to 9703 (Table S1, HILIC negative mode), 8726 (Table S2, HILIC positive mode), 6911 (Table S3, RP negative mode), and 9516 (Table S4, RP positive mode). A manual data curation was then performed by excluding features with standard (SR being the ratio of the intensity in the internal standard sample to that in the biological sample) and blank (BR being the ratio of the intensity in the blank to that in the biological sample) ratio values above 0.15, signal-to-noise ratio (S/N) below 10, relative standard deviation (RSD) exceeding 100% within any individual biological sample group, and RSD greater than 25% in each of the six biological sample groups. Features with SR or BR values > 0.15 were predominantly detected in the internal standard or blank samples, respectively, indicating that they originate from background or carryover rather than from the biological samples. Subsequent amalgamation resulted in a final dataset containing 12,343 features (Table S5).

**Figure 1:**
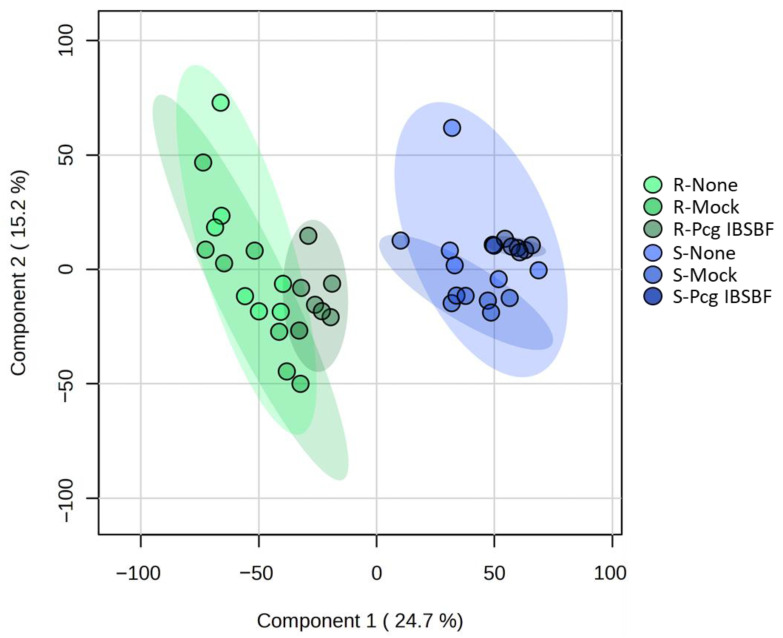
Comparative analysis of leaf metabolome using multivariate statistical approaches. Partial least squares–discriminant analysis (PLS-DA) of untargeted metabolomics was performed without consideration of annotation. Data revealed distinct metabolic differences between the resistant (R) and susceptible (S) *Coffea* leaf accessions along component 1. Group separation was also observed between leaves infected by *Pseudomonas coronafaciens* pv. *garcae* (*Pcg*) (Pcg IBSBF) and uninfected controls (None and Mock) along component 1. Shaded areas in the plot denote 95% confidence intervals (*n* = 6).

Statistical analyses were then conducted on this dataset to identify groups displaying the highest degree of divergence in their metabolic profiles. Principal component analysis (PCA) and partial least squares–discriminant analysis (PLS-DA) were employed to investigate group separation (Figure 1 and Figure S2). In the PCA, the first two principal components (PC) accounted for 44.6% of the total variance, with PC 1 and PC 2 explaining 25% and 19.6%, respectively (Figure S2). In the PLS-DA, the first two components captured a considerable portion of the total variance, collectively accounting for 39.9%. Specifically, components 1 and 2 explained 24.7% and 15.2% of the total variance (Figure 1), respectively. The PLS-DA model incorporating two components revealed improved classification performance compared to the single-component model, achieving accuracy = 0.534, R2 = 0.898, and Q2 = 0.810 (Figure S3). These metrics suggest that the model is both well-fitted and reliable. To assess the statistical robustness of the PLS-DA model, permutation tests (“separation distance” and “prediction accuracy during training”) were then conducted by randomly reassigning class labels over 2000 iterations (Figure S4). The permutation tests yielded null distributions based on 2000 iterations, with test statistics centered around a mean of approximately 0.275 for “prediction accuracy during training” and close to zero for “separation distance” (Figure S4A,B). These distributions reflect the expected outcomes under the null hypothesis, indicating no meaningful discrimination between groups. In contrast, the test statistic value obtained from the original PLS-DA model (observed statistics) was substantially higher than the mean and located in the extreme right tail of the distribution. For the “prediction accuracy during training”, only 51 out of the 2000 iterations produced a test statistic value equal to or greater than the observed statistic, yielding a *p*-value for the permutation test of about 0.0255. For “separation distance”, none of the 2000 permutations produced a test statistic equal to or higher than the observed statistics, yielding a *p*-value < 5 × 10^−4^. A *p*-value < 0.05 for both permutation tests supported null hypothesis rejection and confirmed that the group separation in the PLS-DA score plot reflects biological differences rather than model overfitting.

Both the PCA and PLS-DA models showed a clear separation between the resistant (R) and susceptible (S) accessions under both infected and uninfected conditions. This discrimination occurred along PC 1 in the PCA and the first component in the PLS-DA (Figure 1 and Figure S2). These results suggested that the leaf metabolome is highly different in the resistant compared to the susceptible accessions. The PCA did not reveal any discrimination between infected (Pcg IBSBF) and uninfected (Mock and None) groups (Figure S2). In contrast, discernible shifts were observed between these groups along component 1 of the PLS-DA (Figure 1). However, the overlap of the 95% confidence intervals, depicted as shaded regions in the PLS-DA projection (Figure 1), indicates that infection by *Pcg* induced only modest changes in the metabolome of both susceptible and resistant *Coffea* accessions. These findings point to intrinsic metabolic traits, rather than infection-triggered adjustments, as the main factors underlying the resistance observed in the *Coffea* accessions.

**Figure 2:**
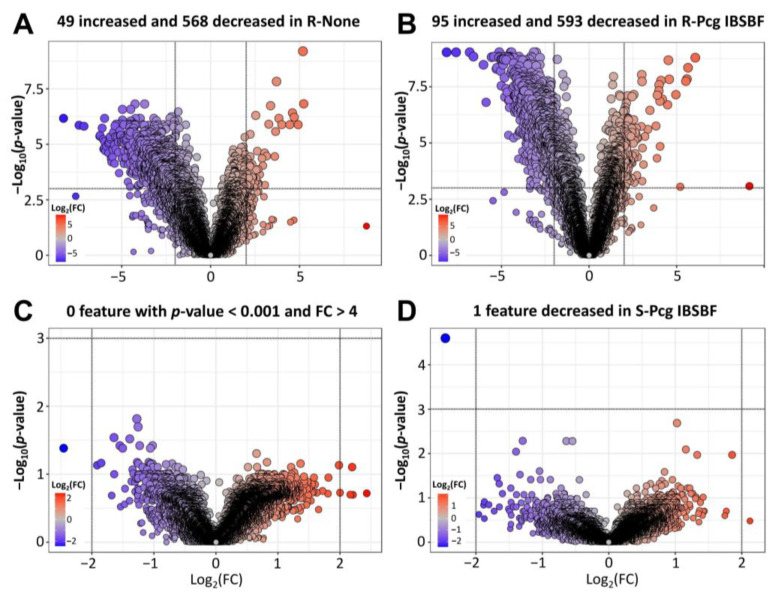
Differential changes in the *Coffea* leaf metabolome illustrated by volcano plots. Volcano plots were constructed from untargeted metabolomic data acquired from *Coffea* leaves without consideration of the annotation. Metabolites with a log_2_ fold change (log_2_(FC)) higher than 2 in absolute value (FC threshold set at 4) and a Student’s *t*-test *p*-value lower than 0.001 were considered significantly altered between two distinct groups. Molecules that are increased (higher log_2_(FC)) are in red, and the ones that are decreased (lower log_2_(FC)) are in blue. The plots show metabolite analysis for (**A**) R-None (uninfected resistant leaves) vs. S-None (uninfected resistant leaves), (**B**) R-Pcg IBSBF (infected resistant leaves) vs. S-Pcg IBSBF (infected susceptible leaves), (**C**) R-Mock (infiltrated resistant leaves) vs. R-Pcg IBSBF (infected resistant leaves), and (**D**) S-Mock (infiltrated susceptible leaves) vs. S-Pcg IBSBF (infected susceptible leaves). The number of features meeting the defined significance criteria (*p*-value < 0.001 and FC > 4) is indicated above each volcano plot (*n* = 6).

To evaluate the number of features with altered accumulation patterns, volcano plot analyses were subsequently performed using fold-change (FC) and *p*-value thresholds of 4 and 0.001, respectively, comparing (i) resistant vs. susceptible accessions (Figure 2A,B) and (ii) infected vs. uninfected leaves (Figure 2C,D). The volcano plots revealed substantial differences in feature accumulation between resistant and susceptible accessions. Under uninfected conditions (Figure 2A), 617 features were differentially accumulated in the resistant leaves compared to the susceptible counterpart, exhibiting an FC greater than four and a *p*-value below 0.001. Among these 617 features, 49 displayed elevated levels, while 568 were reduced in the resistant leaves. Upon infection (Figure 2B), a total of 688 features showed differential accumulation between the resistant and susceptible leaves, defined by an FC exceeding four and a *p*-value lower than 0.001. Of these, 95 exhibited higher levels in the resistant accession, while 593 were less abundant compared to the susceptible counterpart. Conversely, when comparing infected and uninfected leaves, no features met the established significance criteria in the resistant leaves (Figure 2C), while only a single feature exhibited a significant change in the susceptible leaves (Figure 2D). Differences in leaf metabolome between the resistant and susceptible accessions were more prominent than the metabolic shifts induced by *Pcg* infection. These findings indicate that resistance mechanisms appear to be largely pre-established in the resistant *Coffea* accession, rather than being activated only after *Pcg* infection.

### 2.3. Metabolomic Profiling Reveals Biomarkers of Pcg Resistance in Coffea Leaf Tissue

To identify metabolites potentially involved in resistance, feature annotation was performed using in-house and public libraries, focusing on the comparison between the susceptible and resistant leaves under uninfected conditions (S-None vs. R-None). Annotation rates achieved with MS-DIAL 5 were estimated to be 54% for HILIC negative (5251 of 9703 features; Table S6), 83% for HILIC positive (7268 of 8726; Table S7), 50% for RP negative (3471 of 6911; Table S8), and 69% for RP positive (6558 of 9516; Table S9). After removing features considered duplicates across all acquisition modes, as well as excluding those missing MS2 spectra, failing to meet the defined quality thresholds (SR and BR ≤ 0.15, S/N ≥ 10, FC ≥ 4, *p*-value < 0.05, total score ≥ 1, and RSD < 100%), and associated with a long name, the final amalgamated dataset comprised a total of 22 significant and known features (Table S10). In parallel, features were also annotated using MS-FINDER and subsequently filtered based on quality criteria including FC ≥ 4, total score ≥ 5, *p*-value < 0.05, and S/N ≥ 10. After amalgamation, the resulting dataset included seven supplemental known features (Table S11). In total, 29 significant features representing 20 distinct metabolites were retained (Tables S10 and S11 and Figure S5A).

**Figure 3:**
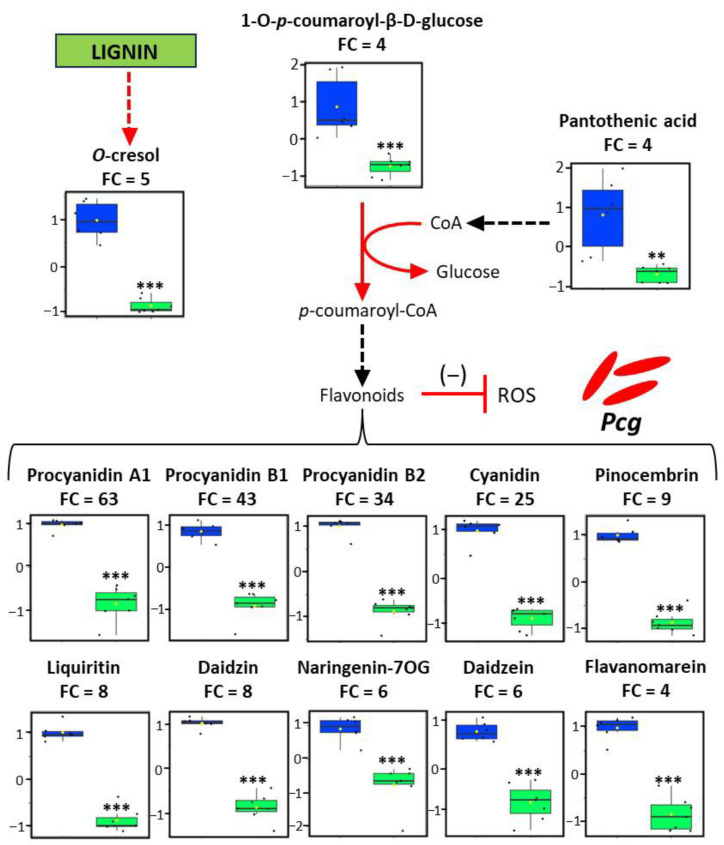
Metabolomic shift reveals differential accumulation of metabolites in the susceptible leaves. Box plots represent the relative abundance of selected metabolites in the susceptible (blue) compared to the resistant (green) accession. These metabolites were mapped into a metabolic and regulatory framework. Solid straight arrows indicate single-step enzymatic reactions, while dashed straight arrows represent multi-step enzymatic conversions. T-bar arrows denote inhibitory regulation. Arrow color reflects confidence in the mechanism: black arrows indicate well-established pathways, whereas red arrows/T-bars suggest putative enzymatic or regulatory interactions. Statistical analysis was performed using a bilateral Student’s *t*-test on unpaired samples (*n* = 6, *p*-value < 0.01: **, *p*-value < 0.001: ***). Black dots represent individual data points. Abbreviation: CoA, coenzyme A; ROS, reactive oxygen species; *Pcg*, *Pseudomonas coronafaciens* pv. *garcae*; naringenin-7OG, naringenin-7-O-glucoside.

**Figure 4:**
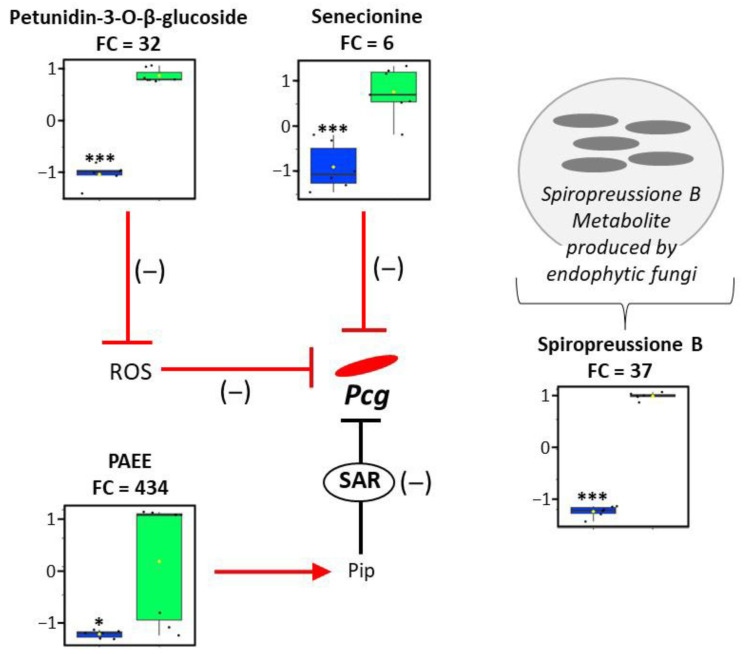
Metabolomic profiling of *Coffea* leaves indicates distinct metabolite accumulation patterns in the resistant accession. Box plots illustrate the relative abundance of metabolites that accumulate in the resistant (green) versus susceptible (blue) *Coffea* accessions. These metabolites were integrated into a metabolic and regulatory network. Solid arrows represent single enzymatic reactions, while T-bar symbols indicate inhibitory regulation. The color of arrows and T-bars reflects the degree of confidence for each pathway: black T-bars correspond to systemically acquired resistance (SAR) mechanisms already described in other plant systems, whereas red symbols denote enzymatic reactions or regulations suggested to occur in the resistant *Coffea* accession. The red arrow specifically marks the putative hydrolysis of pipecolic acid ethyl ester (PAEE) into pipecolic acid (Pip). Statistical significance was assessed using a bilateral Student’s *t*-test (*n* = 6, *p*-value < 0.05: *, *p*-value < 0.001: ***). Black dots correspond to individual data points. Abbreviation: ROS, reactive oxygen species; *Pcg*, *Pseudomonas coronafaciens* pv. *garcae*.

These 20 metabolites were classified into six major superclasses: phenylpropanoids and polyketides (60%), benzenoids (15%), organic oxygen compounds (10%), organic acids and derivatives (5%), alkaloids and derivatives (5%), and organoheterocyclic compounds (5%) (Figure S5A). Within the phenylpropanoids and polyketides superclass, 92% of the compounds were identified as flavonoids (Figure S5B), highlighting a strong enrichment of this subclass. Among the 20 annotated metabolites, a total of 17 were successfully interpreted in a biological context (Figure 3 and Figure 4). The susceptible leaves exhibited elevated levels for 13 metabolites (Figure 3), including 10 reactive oxygen species (ROS)-scavenging flavonoids associated with an FC reaching up to 63-fold. These 10 flavonoids included cyanidin (FC = 25, *p*-value = 3.94 × 10^−8^), daidzein (FC = 6, *p*-value = 3.44 × 10^−6^), daidzin (FC = 8, *p*-value = 2.10 × 10^−8^), flavanomarein (FC = 4, *p*-value = 4.69 × 10^−7^), liquiritin (FC = 8, *p*-value = 9.09 × 10^−9^), naringenin 7-O-glucoside (FC = 6, *p*-value = 0.00013), pinocembrin (FC = 9, *p*-value = 7.73 × 10^−9^), and three procyanidins: A1 (FC = 63, *p*-value = 3.15 × 10^−7^), B1 (FC = 43, *p*-value = 1.95 × 10^−7^), and B2 (FC = 34, *p*-value = 1.75 × 10^−8^). Additionally, the susceptible leaves showed increased levels of flavonoid precursors, including 1-O-p-coumaroyl-beta-D-glucose (FC = 4, *p*-value = 0.00045) and pantothenic acid (FC = 4, *p*-value = 0.00180), and elevated abundance of a potential product of lignin degradation, o-cresol (FC = 5, *p*-value = 2.42 × 10^−7^). The resistant leaves were characterized by the accumulation of four metabolites (Figure 4). Unlike susceptible leaves, only one ROS-scavenging compound, corresponding to petunidin-3-O-beta-glucoside (FC = 32, *p*-value = 2.56 × 10^−8^), was increased in the resistant accession. Instead, the resistant leaves highly accumulated pipecolic acid ethyl ester (a structural derivative of systemic acquired resistance signal, FC = 434, *p*-value = 0.01329), spiropreussione B (endophyte-associated molecule, FC = 37, *p*-value = 6.40 × 10^−14^), and senecionine (potential role in plant defense, FC = 6, *p*-value = 0.00015). The data show that susceptible leaves mainly accumulate flavonoid-type antioxidants (91% showing increased levels; Figure S5C), while resistant leaves are richer in metabolites linked to defense signaling and stress adaptation. Metabolites enriched in the resistant leaves represent potential contributors to the underlying resistance mechanisms.

### 2.4. Pipecolic Acid Ethyl Ester Does Not Directly Influence Pcg Inhibition

Our results showed that pipecolic acid ethyl ester, in contrast to the other metabolites, accumulated to the highest level (434-fold) in the resistant compared to the sensitive accession, suggesting a possible antimicrobial role against *Pcg*. To evaluate this, we conducted an in vitro disk diffusion assay using concentrations of pipecolic acid ethyl ester ranging from 0.1 to 2 mM. Sterile filter-paper disks impregnated with the compound were placed on nutrient agar (NA) medium inoculated with a standardized *Pcg* suspension. After 72 h incubation, no inhibition halo was observed at any concentration tested. These findings indicate that, although this compound is potentially associated with resistance in *Coffea*, it does not directly inhibit *Pcg* growth.

## 3. Discussion

In this study, an untargeted metabolomic approach was employed in leaf tissue from two *Coffea* accessions, one resistant and one susceptible to *Pcg* infection. High-performance liquid chromatography coupled with high-resolution quadrupole–time-of-flight mass spectrometry (HPLC-HR-QTOF) was used to analyze both inherent differences among the accessions and alterations induced by infection. Our analysis aimed to determine whether (i) the resistant accession naturally produces metabolites linked to plant defense and (ii) infection stimulates the production of these compounds.

To identify candidate biomarkers potentially involved in *Pcg* resistance, the metabolome of the resistant *Coffea* leaf accession was first compared to that of the susceptible one. This analysis revealed distinct metabolite profiles between the accessions (Figure 1 and Figure 2A,B). The differences suggest two possible mechanisms: (i) the activation of specific biochemical pathways in the resistant accession that generate protective compounds, or (ii) genetic background differences that could influence biomass-related components and thereby shape the metabolome. However, since no major biomass changes were observed between both accessions (Figure S1), the metabolomic differences (Figure 2A,B) most likely reflect differentially accumulated compounds that contribute to either enhanced or reduced resistance to *Pcg*. Moreover, resistance to the bacterium investigated in this study is known to be governed by the action of a single dominant gene [29]. In this previous research work, all selfed progeny of the susceptible accession IAC 125 RN were susceptible, whereas all selfed progeny of the resistant Ethiopian accession IAC 2211-6 were resistant, demonstrating a clear and stable segregation pattern. This genetic evidence reinforces the interpretation that the observed metabolite profile alterations between accessions are likely related to defense responses rather than genetic background differences. Interestingly, only minor differences in metabolite profile were observed between infected and uninfected leaves (Figure 1 and Figure 2C), implying that plant defense-related mechanisms are likely pre-established in the resistant accession prior to *Pcg* infection rather than being induced in response to pathogen exposure. Additionally, *Pcg* infection caused significant shifts in the biomass composition of the susceptible accession, whereas the resistant leaves maintained stable biochemical profiles (Figure S1)—supporting the idea that defense mechanisms in the resistant genotype prevent pathogen-driven biomass disturbance.

Pipecolic acid ethyl ester was found to accumulate preferentially in the resistant leaf accession of *Coffea*, showing a 434-fold increase compared to its susceptible counterpart (Figure 4 and Table S10). This metabolite is a derivative of pipecolic acid, a compound known to play a key role in systemic acquired resistance (SAR) against pathogen infection [30]. Pipecolic acid modulates the production of free radicals during infection. Specifically, Wang et al. showed that it enhances nitric oxide (NO) biosynthesis, therefore promoting the accumulation of reactive oxygen species (ROS) in locally infected leaves [31]. These ROS subsequently oxidize galactolipid-derived unsaturated C18 fatty acids, namely oleic (C18:1), linoleic (C18:2), and linolenic (C18:3) acids, leading to the production of azelaic acid, a C9 dicarboxylic acid. Azelaic acid stimulates the biosynthesis of glycerol-3-phosphate (G3P), which is translocated to distal leaves to further induce ROS production. These events define a signaling cascade where ROS, and by extension pipecolic acid, function as crucial mediators of defense. These molecules suppress pathogen proliferation and fortify plant resistance against biotic stresses [31,32]. Interestingly, rather than elevated levels of pipecolic acid, our analysis revealed a sharp enrichment of its ethyl ester form in the resistant *Coffea* leaves. While the function of pipecolic acid ethyl ester in plants has not been characterized, certain esters of related SAR-associated metabolites—such as the methyl ester of N-hydroxypipecolic acid—have been shown to potentially act as a transport or storage form of N-hydroxypipecolic acid that can be hydrolyzed to release the active free compound involved in plant immunity [33]. Whether a similar mechanism applies to pipecolic acid ethyl ester in *Coffea* remains to be determined. To explore whether this compound exhibits direct antimicrobial activity, we performed an in vitro disk diffusion assay using various concentrations of pipecolic acid ethyl ester (0.1–2 mM) standard. No inhibition halos were observed, aligning with previous studies indicating that pipecolic acid and N-hydroxypipecolic acid primarily function as immune regulators rather than direct antibacterial agents [34,35,36]. While these findings do not establish a functional role for pipecolic acid ethyl ester in *Coffea* immunity, they provide a basis for future investigations into its potential involvement in defense-related metabolic pathways.

The resistant accession also exhibited an increase in a single anthocyanin, petunidin-3-O-β-glucoside (Figure 4 and Table S10). Anthocyanins have been widely described as antioxidants [37], with roles in neutralizing ROS, limiting their formation, or reducing their reactivity [38]. As mentioned above, ROS are central to plant immune responses, but their excessive accumulation can cause cellular damage, including membrane disruption, enzyme inactivation, and DNA degradation [32]. The higher abundance of this unique antioxidant compound in the resistant accession may contribute to moderating ROS accumulation without markedly affecting ROS-dependent defense functions. In contrast, the susceptible leaves displayed a distinct metabolic profile, characterized by the accumulation of multiple flavonoids and precursors. Indeed, our findings showed an increase in pantothenic acid and 1-O-p-coumaroyl-β-D-glucose in this accession (Figure 3 and Table S11). Pantothenic acid, synthesized endogenously in higher plants [39], serves as a precursor to coenzyme A (CoA) [40,41], which in turn may facilitate the conversion of 1-O-p-coumaroyl-β-D-glucose into p-coumaroyl-CoA, a critical intermediate in flavonoid biosynthesis [42]. A total of 10 flavonoids was accumulated in the susceptible accession (Figure 3 and Table S10), including proanthocyanidins (procyanidin A1, B1, and B2), anthocyanidins (cyanidin), flavanones (pinocembrin, flavanomarein, and naringenin 7-O-glucoside), flavones (liquiritin), and isoflavones (daidzin and daidzein). These 10 compounds have well-documented antioxidant activity [43,44,45,46,47,48,49,50,51,52], and their elevated abundance may be associated with a stronger reduction in ROS levels, a pattern consistent with the higher susceptibility observed in this accession. Whether the differential accumulation of these antioxidants in the resistant and susceptible *Coffea* leaves is associated with maintained or altered ROS homeostasis still needs to be clarified.

Additionally, our findings revealed elevated levels of spiropreussione B and senecionine—two bioactive compounds known for their roles in plant immunity through the inhibition of pathogenic growth—in the resistant accession (Figure 4 and Tables S10 and S11). Spiropreussione B, in particular, is produced by endophytic fungi of the *Preussia* genus, which have previously been identified in *Coffea* plants [53,54]. Endophytic fungi are symbiotic microorganisms that reside within plant tissues without inducing disease while enhancing host resilience by synthesizing antimicrobial secondary metabolites [55]. The higher abundance of spiropreussione B may reflect enhanced endophytic colonization and/or metabolic exchange with beneficial microbes, a hypothesis that warrants microbiome-level validation. In contrast, the lower abundance observed in the susceptible accession may indicate altered endophytic colonization. This differential colonization may represent a key factor in enhancing resistance to phytopathogenic infections, positioning endophytic fungi as essential contributors to *Coffea*’s defense mechanisms. While spiropreussione B suggests the contribution of endophytic fungi to *Coffea*’s defense, senecionine, as a pyrrolizidine alkaloid, might represent an additional layer of protection derived directly from the plant. Pyrrolizidine alkaloids have been shown to inhibit bacterial and fungal growth and suppress the expression of virulence genes [56,57], suggesting a role for senecionine in limiting *Pcg* proliferation and virulence.

Finally, our results revealed a higher abundance of *o*-cresol in the susceptible accession compared to its resistant counterpart (Figure 3 and Table S11). *O*-cresol is a naturally occurring compound in plants [58], and it can also be formed through lignin degradation under extreme conditions [59]. However, the bioconversion of lignin into *o*-cresol via biological pathways remains largely unexplored. To the best of our knowledge, lignin degradation typically yields syringyl, guaiacyl, and *p*-hydroxyphenyl units [60], which are structurally related phenolic derivatives, like *o*-cresol. Considering the structural role of lignin in cell wall reinforcement and its function as a physical barrier against phytopathogen invasion [61], its potential reduction could contribute to the increased susceptibility of *Coffea* plants to *Pcg* infection. Whether lignin is ultimately affected in the susceptible leaves still remains to be elucidated.

In conclusion, our findings identified several candidates potentially associated with defense responses in the resistant *Coffea* accession. The resistant leaves significantly accumulate pipecolic acid ethyl ester, a structural derivative of pipecolic acid, itself a well-recognized regulator of plant defense. Although the biological activity of the ethyl ester form has not yet been characterized, its accumulation in the resistant accession may point to upstream metabolic configurations associated with defense priming. This interpretation aligns with the proposed role of other esterified SAR-related molecules, such as N-hydroxypipecolic acid methyl ester, which can function as a storage or transport intermediate and be hydrolyzed to release free N-hydroxypipecolic acid, a compound known to participate in plant immune regulation. Beyond this, the two *Coffea* accessions differed markedly in their antioxidant profiles. The resistant accession only accumulates petunidin 3-O-β-glucoside, whereas the susceptible leaves displayed a broad induction of flavonoids. Due to the ROS-quenching activity of these compounds, their elevated levels in the susceptible accession may excessively reduce ROS and compromise the activation of effective defense responses. However, this interpretation still requires further experimental confirmation. Additionally, the presence of antimicrobial compounds in the resistant accession, such as senecionine and spiropreussione B, underscores the role of both plant-derived and endophyte-associated metabolites in pathogen suppression or virulence attenuation. Together, these findings indicate that *Coffea* resistance relies not only on constitutive metabolite accumulation but also on synergistic interactions with beneficial endophytes, forming a multi-layered defense system. Understanding these preformed and microbially reinforced metabolic defenses provides new perspectives for breeding or engineering *Coffea* plants with durable resistance to *Pcg*.

## 4. Materials and Methods

### 4.1. Chemical and Reagent

Reagents and chemicals used for fatty acid extraction, methylation, and quantification, which include glyceryl triheptadecanoate (C17:0 TG), toluene, sulfuric acid, and sodium hydrogen sulfate (NaHSO_4_), were from Sigma-Aldrich (St. Louis, MO, USA), whereas hexane, isopropanol, and methanol were purchased from Thermo Fisher Scientific (Waltham, MA, USA). A buffer composed of Tris(hydroxymethyl)aminomethane (Tris-HCl), sodium chloride (NaCl), and sodium dodecyl sulfate (SDS) was ordered from Thermo Fisher Scientific (Waltham, MA, USA), while DC Protein Assay Reagents were procured from Bio-Rad (Hercules, CA, USA). Glacial acetic acid from Sigma-Aldrich (St. Louis, MO, USA) and the total starch assay kit from Megazyme (Bray, Ireland) were used for the extraction and quantification of starch. For untargeted metabolomics, ^13^C-glycine, ^13^C-fumarate, and ^13^C-benzoic acid internal standards were provided by Cambridge Isotope Laboratories (Tewksbury, MA, USA). Metabolite standards were obtained from different companies, including Thermo Fisher Scientific, Sigma-Aldrich, Toronto Research Chemicals, Cayman Chemical, BOC Sciences, and Benchchem. Solvents and additives, like acetonitrile, water, ammonium formate, and formic acid, used for GC-MS and LC-MS/MS analyses, were procured from Thermo Fisher Scientific (Waltham, MA, USA).

### 4.2. Plant Cultivation, Infiltration, and Leaf Infection by Pcg

The study was conducted on coffee plants approximately four years old, whose disease responses had been previously characterized [29]. Plants were grown in pots under controlled greenhouse conditions. Two genotypes were selected: the resistant IAC 2211-6 accession from the FAO Coffee Mission to Ethiopia (1964–1965) [62] and the susceptible cultivar IAC 125 RN. The sampling procedure was standardized by consistently selecting the first two pairs of fully expanded leaves counted from the shoot apex. This sampling strategy was adopted to reduce metabolomic variability associated with leaf developmental stage and to ensure greater consistency across treatments. The inocula for plant inoculations were prepared from bacterial colonies of the *Pcg* strain IBSBF 1197, which is highly aggressive in coffee plants [63]. Bacterial cultures were grown on nutrient agar media for 48 h until reaching the logarithmic growth phase. The colonies were then suspended in sterilized distilled water, and the bacterial concentration was adjusted to approximately 10^8^ CFU mL^−1^ [64] using a spectrophotometer (OD = 0.3). Inoculations were subsequently performed by infiltrating the bacterial suspension into the leaf lamina [65]. Two control treatments were established: leaves infiltrated with sterile distilled water and healthy plants without any wounds from the infiltration process. These controls were designed to eliminate potential responses not directly associated with bacterial infection. The experimental design included six leaves per treatment. After infiltration, the plants were maintained in the laboratory under controlled conditions until tissue collection, which occurred 24 h post-infiltration with Pcg IBSBF 1197 or the respective control treatments. The leaves were harvested and rapidly immersed in liquid nitrogen to preserve their metabolic profiles. The samples were freeze-dried in a lyophilizer (Christ, alpha 2-4 LD Plus) at a temperature of −70 °C, also for 96 h, and subsequently prepared for metabolomics analysis.

### 4.3. Biomass Extraction and Quantification

The biomass extraction protocol was adapted to coffee leaves based on previously published methods [66,67]. Biomass extraction was achieved on 10 mg of dried ground coffee leaves previously lyophilized for 4–5 days. The lipid extraction was first performed under a biosafety cabinet. Fifty µL of a previously warmed glyceryl triheptadecanoate (C17:0 TG, 1 mg/mL, Sigma-Aldrich, MO, USA) internal standard was added to biological samples containing 950 µL of hexane/isopropanol (2:1, *v*/*v*). The lipid extract was disrupted for 5 min using a 5 mm tungsten bead with a Retsch Mill MM 400 bead beater (Retsch, Haan, Germany). Following, samples were centrifuged at 17,000× *g* for 15 min at room temperature, and the resulting lipid-containing supernatant was then transferred to a 13 × 100 mm glass screw-cap tube. For better recovery, the protocol was repeated twice using 1 mL and then 0.5 mL of the hexane/isopropanol (2:1, *v*/*v*) mix for the second and third lipid extractions, respectively. The combined lipid extract was centrifuged at 800× *g* for 5 min at room temperature (Swinging bucket Legend X1R, Thermo Scientific, Waltham, MA, USA). The lipid-containing supernatant was then transferred to a new 13 × 100 mm glass tube, evaporated under a nitrogen stream, resuspended in 150 µL toluene, and vortexed for 5–10 s. A volume of 0.5 mL of a freshly prepared methylation mix (2.5% sulfuric acid (*v*/*v*) in methanol) was added to the lipid extract, which was vortexed for 30 s. After 90 min incubation at 80 °C, fatty acid methyl esters (FAMEs) were cooled down at room temperature for 3 min before adding 250 µL of a quenching solution (5% NaHSO_4_ in H_2_O) and 1 mL of hexane. The FAME extracts were vigorously vortexed for 1 min and then centrifuged at 800× *g* for 5 min at room temperature in a swinging bucket (Legend X1R). Then, 500 µL of the organic upper phase was transferred to a new 13 × 100 mm glass tube, evaporated under a nitrogen stream, and resuspended in 200 µL of hexane. The FAME extracts were then added to a 250 µL glass insert placed in a 2 mL screw-capped glass vial (Thermo Scientific, Waltham, MA, USA). FAMEs were analyzed and quantified by GC-MS (6890N Network GC system and a single-quadrupole 5975B VLMSD, Agilent Technologies, Santa Clara, CA, USA). GC-MS conditions used for the analysis were previously described [68,69]. Protein extraction was performed on the remaining pellet depleted of fatty acids. In brief, 0.5 mL of a protein extraction buffer, constituted of 20 mM Tris-HCl (pH 7.5), 150 mM NaCl, and 1% SDS, and warmed to 42 °C, was added to the defatted extracts. Samples were incubated in a thermomixer at 1500 rpm for 15 min at 42 °C and then centrifuged at 17,000× *g* for 10 min at room temperature. Supernatants were transferred into new 2 mL microcentrifuge tubes (USA Scientific, Ocala, FL, USA), and the steps described above were repeated twice to recover additional proteins. Total protein content was determined using the DC Protein Assay kit (Bio-Rad: Hercules, CA, USA) and the Bio-Rad SmartSpec Plus spectrophotometer (Bio-Rad: Hercules, CA, USA) at 750 nm [69]. For starch extraction, 1 mL of distilled water was added to protein-depleted pellets. Samples were vortexed, centrifuged at 17,000× *g* for 10 min at room temperature, and supernatants were discarded. The steps described above were repeated by adding 1.5 mL of distilled water to the pellet. After removing the supernatant, 0.5 mL of 0.1 M acetate buffer, pH 4.8, was added to the pellets. Samples were autoclaved at 120 °C for one hour and then cooled down for 15 min at room temperature. A volume of 0.5 mL of 0.1 M acetate buffer was added to the samples, and pellets were resuspended using a vortex. The non-soluble starch was digested with 10 µL of amyloglucosidase (Megazyme International Ireland Ltd. Total Starch Assay Kit, Wicklow, Ireland) for 2 h at 55 °C in a dry bath. Samples were mixed by inverting tubes every 30 min during the 2 h incubation time and centrifuged at 17,000× *g* for 15 min at room temperature. Supernatant was transferred into a new 1.5 mL microcentrifuge tube. One mL of GOPOD reagent (Megazyme International Ireland) was added to 35 µL of extract to quantify glucosyl moieties. Absorbance was measured at 510 nm using a Bio-Rad SmartSpec Plus spectrophotometer at 510 nm. The cell wall quantity was determined by subtracting fatty acid, protein, and starch content from leaf dry weight.

### 4.4. Metabolite Extraction and Resuspension

To perform untargeted metabolomics, an adapted chloroform/methanol/water extraction was performed on 10 mg of dried and ground coffee leaves, as previously described [70]. Each sample was transferred to a 2 mL screw-cap plastic tube (Fisher Scientific, Hampton, NH, USA) and supplemented with 1 mL of a chloroform/methanol/water mixture (1:2.5:1, *v*/*v*/*v*) containing 300 nmol of ^13^C-glycine. Samples were homogenized at 4 °C for 10 min at 1500 rpm using a Thermomixer C (Eppendorf, Hauppauge, NY, USA), followed by centrifugation at 17,000× *g* for 5 min at 4 °C. The resulting supernatants were transferred to new 2 mL microcentrifuge tubes (USA Scientific, Ocala, FL, USA), and 400 µL of cold water was added to each. After vortexing, samples were centrifuged again under the same conditions. One mL of the upper aqueous phase was collected and transferred to a 1.5 mL microcentrifuge tube, then divided into two equal aliquots of 500 µL to enable parallel analysis of semi-polar and polar metabolites via LC-MS/MS. Prior to LC-MS/MS analysis, methanol was removed using a SpeedVac concentrator set at 30 °C for 20 min (Savant ISS110 SpeedVac Concentrator, Thermo Scientific, Waltham, MA, USA). Samples were flash-frozen in liquid nitrogen and subsequently lyophilized at −83 °C for 3–4 h to eliminate residual water. Internal standards were prepared in two distinct solvent systems—methanol/water (20:80, *v*/*v*) and acetonitrile/water (80:20, *v*/*v*)—and included 2 nmol each of MOPS, ampicillin, and 9-phenanthrol; 1 nmol of trans-zeatin-d_5_; 50 nmol of ^13^C-fumarate; and 10 nmol each of ^13^C-benzoic acid, fluorocytosine, and trans-cinnamic acid-d_6_. One set of dried aliquots was reconstituted in 100 µL of the methanol-based solution, while the remaining were resuspended in 100 µL of the acetonitrile-based solution. All samples were sonicated for 5 min at 25 °C, incubated in a thermomixer at 1750 rpm for 5 min at 25 °C, and centrifuged at 17,000× *g* for 5 min at 25 °C. Subsequently, 100 µL of each extract was transferred to LC-MS/MS glass vials containing inserts (Agilent, CA, USA). A quality control (QC) was prepared by combining 2 µL from each extract sample into a single LC-MS/MS vial. Blank samples consisted solely of methanol/H_2_O (20/80, *v*/*v*) or acetonitrile/H_2_O (80/20, *v*/*v*) mix. Samples reconstituted in methanol/water were designated for RP analysis, while those resuspended in acetonitrile/water were used for HILIC analysis. Three and 4 µL of the extract, QC, and blanks were injected into the LC-MS/MS equipped with the RP and HILIC columns, respectively.

### 4.5. Analysis by UHPLC-HRMS Triple TOF

Metabolites were analyzed using an ultra-high-performance liquid chromatography (UHPLC) system coupled with a high-resolution mass spectrometer (HRMS) (Exion LC and Triple Time-Of-Flight TOF 6600+, AB Sciex, Framingham, MA, USA). The UHPLC-HRMS system was configured with either an RP (Kinetex F5, 150 × 2.1 mm, 2.6 µm; guard column F5, 10 × 2.1 mm; Phenomenex, Torrance, CA, USA) or a HILIC (ACQUITY Premier BEH Amide VanGuard FIT column: 150 × 2.1 mm, 1.7 µm; ACQUITY Premier BEH Amide pre-column: 5 × 2.1 mm, 1.7 µm; Waters, Milford, MA, USA) pre-column and column to enable complementary chromatographic separation strategies, thereby improving the coverage of plant metabolites during subsequent detection. The autosampler temperature was held at 10 °C, regardless of the chromatographic column employed. The temperature for the RP and HILIC columns was maintained at 20 °C and 35 °C, respectively. The elution of metabolites through the RP column was performed at a flow rate of 0.2 mL/min using a gradient system consisting of solvent A (0.25% *v*/*v* of formic acid and 5 mM ammonium formate in water) and B (0.1% formic acid in acetonitrile, *v*/*v*). The gradient profile for RP was set as described: 0–2.0 min, 0% B; 2.0–18.0 min, 0–80% B; 18.0–18.1 min, 80–95% B; 18.1–21.0 min, 95% B; 21.0–21.1 min, 95–0% B; and 21.1–25.0 min, 0% B. For HILIC, a flow rate of 0.3 mL/min was applied using a gradient system comprising solvent A (0.2% *v*/*v* formic acid and 25 mM ammonium formate in water) and solvent B (0.15% *v*/*v* formic acid and 10 mM ammonium formate in 90% aqueous acetonitrile). The gradient program for HILIC was applied as defined: 0–2.00 min, 100% B; 2.00–6.00 min, 100–70% B; 6.00–9.35 min, 70–40% B; 9.35–11.00 min, 40–30% B; 11.00–13.50 min, 30–100% B; and 13.50–20.00 min, 100% B. Following the separation by UHPLC, a sequential window acquisition of all theoretical mass spectra (SWATH-MS) scan survey was employed as a data-independent acquisition. To define precursor ion windows, data-dependent acquisition scans were first conducted on QC samples under both positive and negative ionization modes for RP and HILIC. This approach enabled the generation of 36 variable SWATH-MS windows targeting precursor ions across an *m*/*z* range of 50–1250 amu, with each scan accumulated over 200 ms. Fragment ion spectra (MS/MS) were collected within an *m*/*z* range of 30–1250 amu, using a 25 ms accumulation time per scan, resulting in a total cycling time of 1.15 s. Instrument parameters associated with the electrospray ionization (ESI) source and quadrupole settings were carefully optimized to ensure robust ionization and transmission/fragmentation of ions across the MS analyzer. Curtain gas pressure was consistently maintained at 35 psi across both RP and HILIC modes. Source temperature settings differed, with 600 °C applied for HILIC and 550 °C for RP. IonSpray voltages were adjusted according to polarity, ranging from −3500 V (HILIC) to −4500 V (RP) in negative mode and 3500 V (HILIC) to 5000 V (RP) in positive mode. Nebulizing and heating gases were adjusted within ranges of 50–60 psi and 60–80 psi, respectively, depending on the chromatographic and ionization mode. Declustering potential was set at ±50 V, and collision energy potential at ±35 V, with a consistent collision energy spread of 10 V applied across all modes to enhance fragmentation reproducibility. To correct for potential mass shift during LC-MS/MS analysis, an atmospheric-pressure chemical ionization (APCI) calibrant solution was introduced every eight samples—alternating between positive and negative modes. Data acquisition was achieved using Analyst TF version 1.8.1 (AB Sciex, Framingham, MA, USA).

### 4.6. Processing of LC-MS/MS Data Using MS-DIAL

Data were processed using MS-DIAL 5 software. Data files generated with Analyst TF version 1.8.1 (.wiff) were imported into MS-DIAL, and SWATH was selected as the acquisition method for each sample. MS1 and MS2 tolerances were set to 0.01 and 0.025 Da, respectively. Retention time windows were optimized to reflect the chromatographic characteristics of both HILIC (1–20 min) and RP (1–18 min) separation modes. Mass ranges for MS1 and MS/MS scans were defined as *m*/*z* = 50–1250 amu and *m*/*z* = 30–1250 amu, respectively. Retention time correction using an internal standard was enabled. Peak detection parameters included a minimum peak height of 1000, a mass slice width of 0.1, and a linear weighted moving average smoothing method with a smoothing level set at three. Peaks with a minimum width of five scans were retained. Deconvolution was performed using a sigma window value of 0.5, with exclusion of signals following the precursor ion and retention of isotopic ions up to 0.5. MS/MS abundance cutoff was set to zero. Several spectral libraries were used to identify/annotate compounds, including an in-house database, MassBank of North America (MoNA), and RIKEN Tandem Mass Spectral Database for Phytochemicals. Identification parameters included mass tolerances of 0.01 Da (MS1) and 0.025 Da (MS2), retention time tolerances of 1 min for the in-house library and 100 min for public libraries, and dot product scores (direct, weighted, and reverse) of 0.5. Annotation was performed when an experimental spectrum matched at least one reference spectrum, with a matched spectrum percentage exceeding 20 and 30% for the in-house and public libraries, respectively. For the in-house library, retention time was used for scoring. Adduct ions included [M + H]^+^, [M + Na]^+^, [M + K]^+^, [M + H-H_2_O]^+^, [M + H-2H_2_O]^+^, and [2M + H]^+^ for positive mode and [M-H]^−^, [M-H_2_O-H]^−^, [M + Na-2H]^−^, [M + FA-H]^−^, [2M-H]^−^, and [2M + FA-H]^−^ for negative mode. The QC sample acquired in the middle of the sequence was used for peak alignment. Retention time and MS1 tolerances were set to 0.1 min and 0.015 Da, respectively. Features present in blanks (sample max/blank average < 5) were removed. Only features matched to reference spectra were retained, while suggested features lacking MS2 data were excluded.

For automatic LC-MS/MS data curation, the MS-CleanR module of MS-DIAL 5 was used to eliminate ghost peaks, as well as features with incorrect *m*/*z* values, high relative standard deviation (RSD ≥ 25%), and those detected in the blanks (blank ratio ≥ 0.15) [71]. Peak integration and alignment were manually reviewed. The raw data obtained from MS-DIAL were subsequently exported into an Excel file to proceed to manual data curation and amalgamation. Manual data curation and amalgamation were initially conducted without consideration of the annotation status. Features were excluded from the original dataset if they met any of the following criteria: standard (SR) and blank (BR) ratios exceeding 0.15, signal-to-noise ratio (S/N) below 10, relative standard deviation (RSD) over 100% within an individual biological sample group, and RSD above 25% in each of the six biological sample groups. Statistical analyses were then conducted to identify groups exhibiting maximal separation within a two-dimensional projection and estimate the number of differentially accumulated features. Subsequent data curation and amalgamation were carried out, incorporating annotation and considering R-None and S-None samples. Duplicate and unidentified features, as well as those lacking MS2 data, failing to meet quality thresholds (SR and BR < 0.15, S/N > 10, FC > 4, *p*-value < 0.05, and total score > 1), and associated with long-name metabolites, were excluded from the datasheet. Annotated features with RSD below 100% were retained.

### 4.7. Annotation Using MS-FINDER

After processing data using MS-DIAL 5, each feature detected in its respective acquisition mode (HILIC, RP, and negative/positive ionization) was individually exported as an .msp peak file. These files were subsequently imported to MS-FINDER v3.72 for tentative structure assignment of unknown compounds [72]. Within MS-FINDER, the following databases—HMDB, YMDB, UNPD, PlantCyc, ChEBI, NPA, NANPDB, COCONUT, KNApSAcK, and PubChem—were selected to enable structure-based annotation. MS-FINDER generated predicted structures for each feature, accompanied by a scoring system ranging from 0 to 10. To ensure consistency, the total score obtained from MS-DIAL was normalized to a maximum of 10, resulting in MS-DIAL total scores also ranging from 0 to 10. Once generated, the MS-FINDER output was manually merged with the original MS-DIAL Excel file using a common identifier, including the alignment ID, average *m*/*z*, metabolite name, adduct type, formula, ontology, INCHIKEY, SMILES, and total score, thereby linking the feature peak height obtained from MS-DIAL with the annotation derived from MS-FINDER. This procedure was repeated for each acquisition mode prior to data amalgamation. Annotated features with FC < 4, total score < 5, *p*-value > 0.05, and S/N < 10 were then excluded from the MS-FINDER Excel dataset.

### 4.8. Statistical Analyses

Statistical analyses were performed using MetaboAnalyst 6.0 [73]. Prior to statistical analyses, data were log_10_-transformed and auto-scaled. Volcano plots, box plots, PCA, and PLS-DA score plots were performed. PCA and PLS-DA were performed using the first two principal components and components, respectively. Both cross-validation (5-fold CV, maximum components to search = 2) and permutation tests (“separation distance” and “prediction accuracy during training”; permutation number = 2000) were performed to confirm the validity and robustness of the PLS-DA model. For the volcano plot, thresholds for the *p*-value (false discovery rate; FDR) and FC were defined as 0.001 and 4, respectively, with group variance assumed to be unequal. Student’s *t*-test and ANOVA Tukey’s test were also used to compare different experimental conditions.

## Data Availability

The original contributions presented in this study are included in the article/Supplementary Materials. Further inquiries can be directed to the corresponding author.

## Supplementary Materials

The following supporting information can be downloaded at https://www.mdpi.com/article/10.3390/plants15020216/s1, Figure S1: Comparative analysis of the biomass composition in *Coffea* leaves (Word document); Figure S2: Comparison of the leaf metabolome using Principal Component Analysis (Word document); Figure S3: PLS-DA cross-validation (Word document); Figure S4: PLS-DA permutation tests assessing model robustness (Word document); Figure S5: Classification and distribution of significantly altered metabolites among *Coffea* accessions (Word document); Supplemental Tables (Tables S1–S11): Untargeted metabolomic LC-MS/MS dataset acquired from *Coffea* leaves (Excel).
