# Supplementary material for: Comparative Metabolomic Profiling of Resistant and Susceptible Coffea arabica Accessions to Bacterial Pathogen Infection
Source: Plants (Basel). 2026 Jan 9;15(2):216. doi: 10.3390/plants15020216 (PMC12844768; doi:10.3390/plants15020216)
Supplement: Supplementary file 1 [file plants-15-00216-s001.zip › Supplemental Figure plants-4055521.pdf]

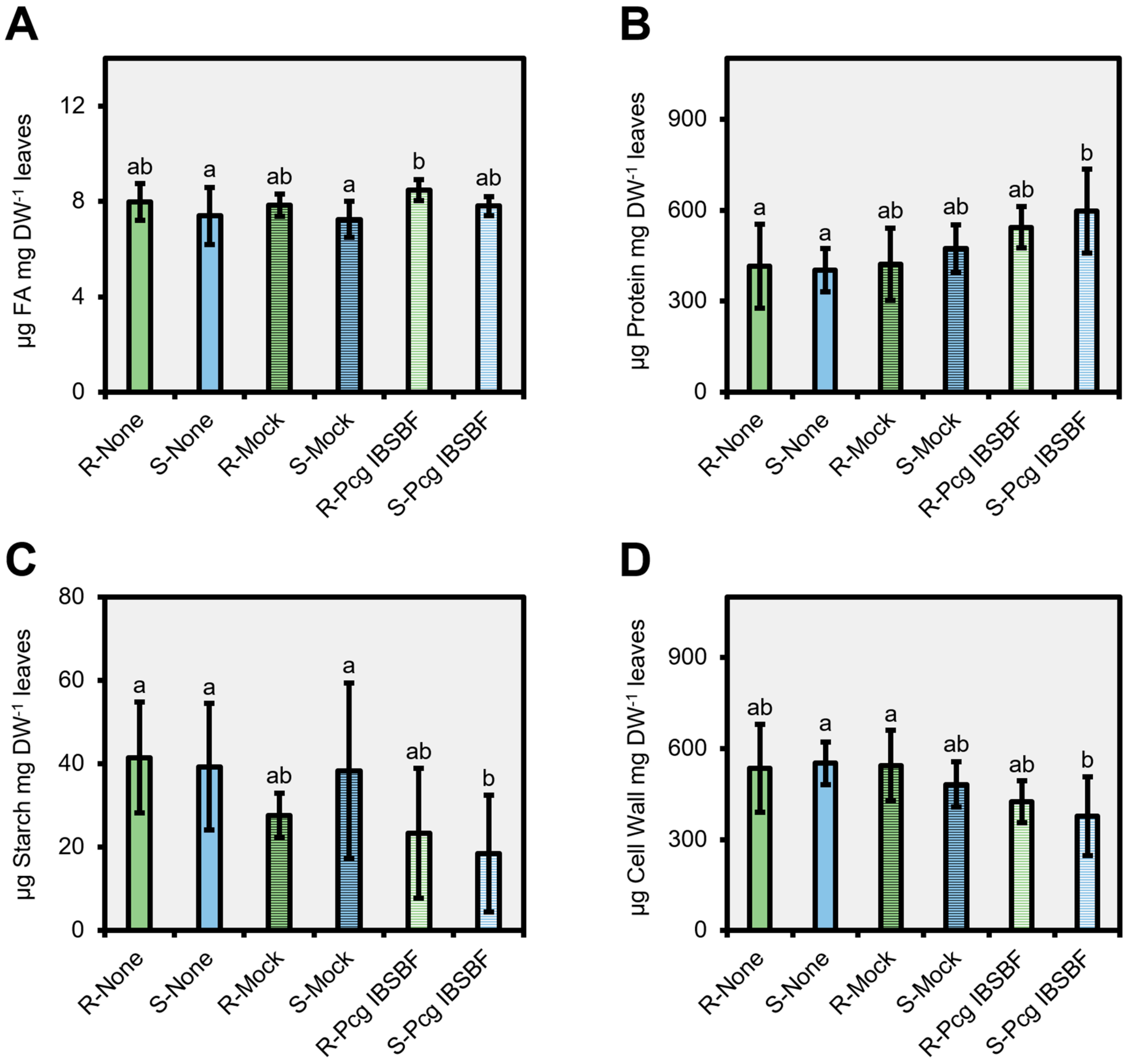

Figure S1. Comparative analysis of the biomass composition in *Coffea* leaves. The total amount (expressed in µg/mg of dry weight leaves) of (A) fatty acids (FA), (B) protein, (C) starch, and (D) cell wall was compared between the susceptible (S) Arabica cultivar (IAC 125 RN) and the resistant (R) Ethiopian accession (IAC 2211-6). The biomass of coffee leaves infected by *Pcg* (Pcg IBSBF) was also compared with the non-infiltrated leaves (None) and leaves infiltrated with water (Mock). Standard deviation corresponds to a number of biological replicates of n=8 for each condition. Bars with different letters are significantly different according to one-way ANOVA Tukey's test (p-value < 0.05).

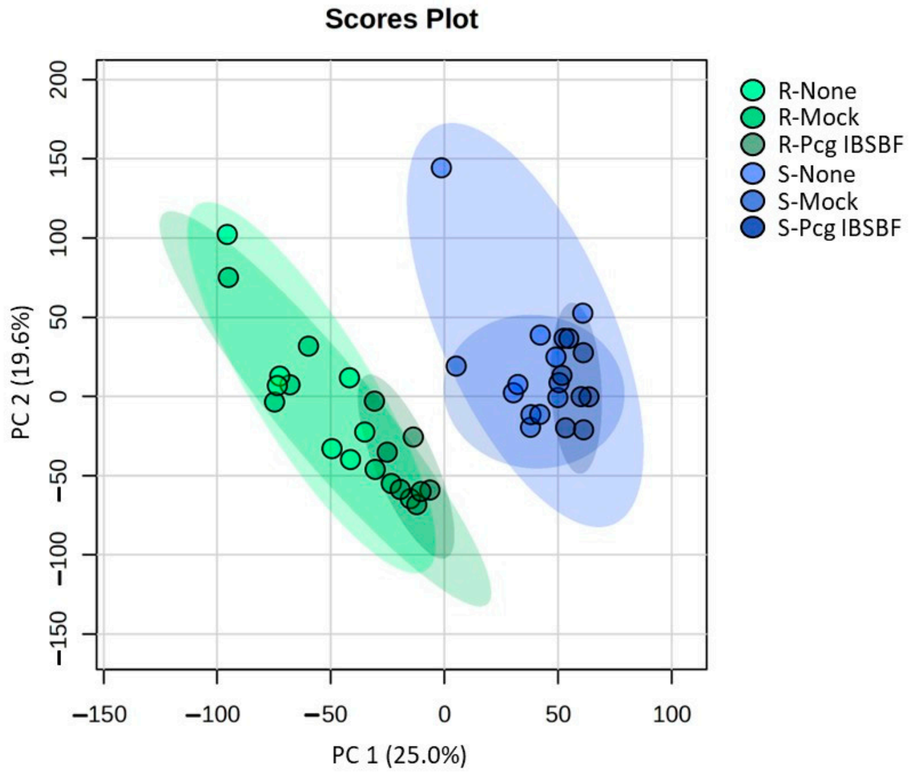

Figure S2. Comparison of the leaf metabolome using Principal Component Analysis. Principal Component Analysis (PCA) of untargeted metabolomic dataset was performed using the first two principal components (PC 1 and PC 2) and without consideration of metabolite annotation. The PCA revealed clear metabolic distinction between resistant (R) and susceptible (S) *Coffea* accessions along PC 1. In contrast, no group separation was observed between infected (Pcg IBSBF) and uninfected controls (None and Mock). Shaded areas denote 95% confidence intervals (n = 6).

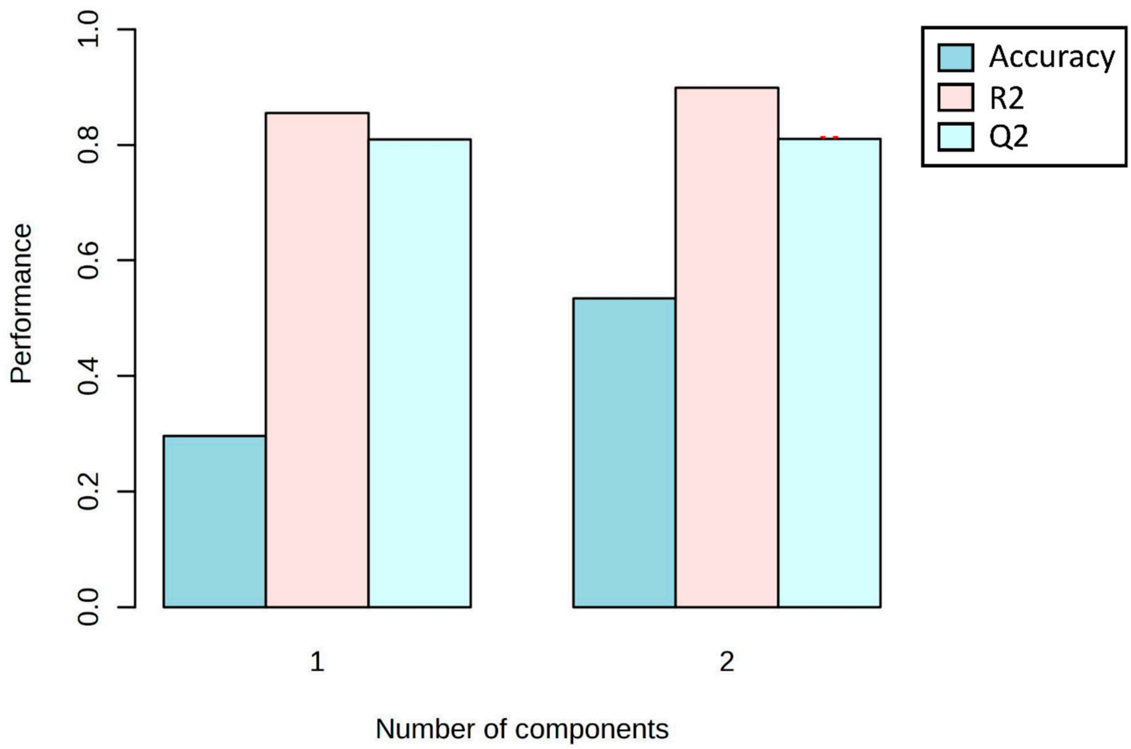

Figure S3. PLS-DA cross-validation. Models were constructed using either one or two components to assess the classification performance. Bar plots display the mean performance scores for each metric (accuracy, R2 and Q2) across the specified number of components. The plot was performed using MetaboAnalyst 6.0.

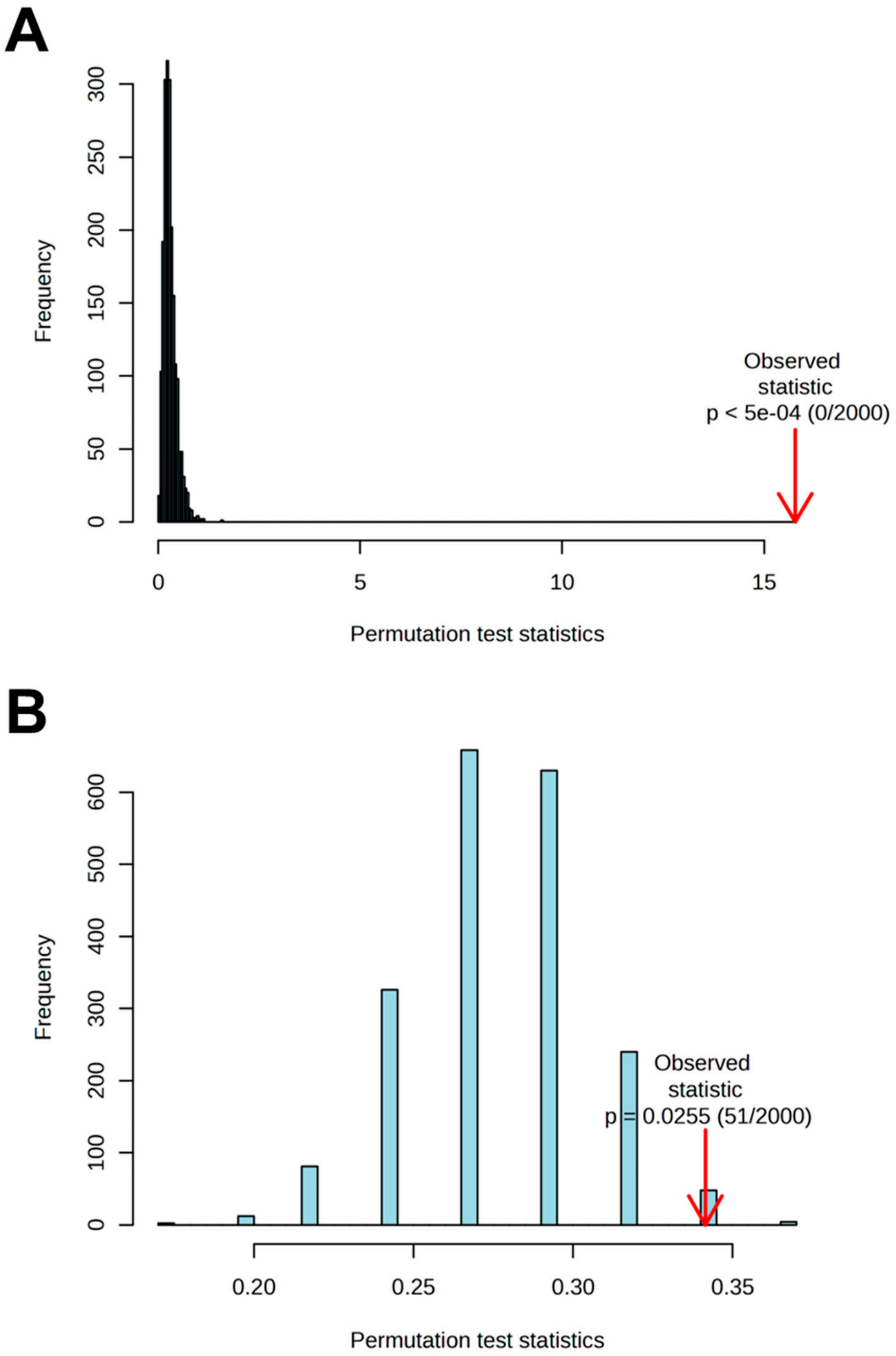

Figure S4. PLS-DA permutation tests assessing model robustness. Permutation tests were performed using MetaboAnalyst 6.0. Two statistical metrics were selected: (A) separation distance (between/within groups, B/W) and (B) prediction accuracy during training. A total of 2,000 permutations were conducted. A permutation test p-value (A) lower than 5×10^{-4} and (B) equal to 0.0255 were obtained using both permutation test methods, respectively. These results indicate statistically significant group separation and predictive performance, supporting the validity of the PLS-DA model.

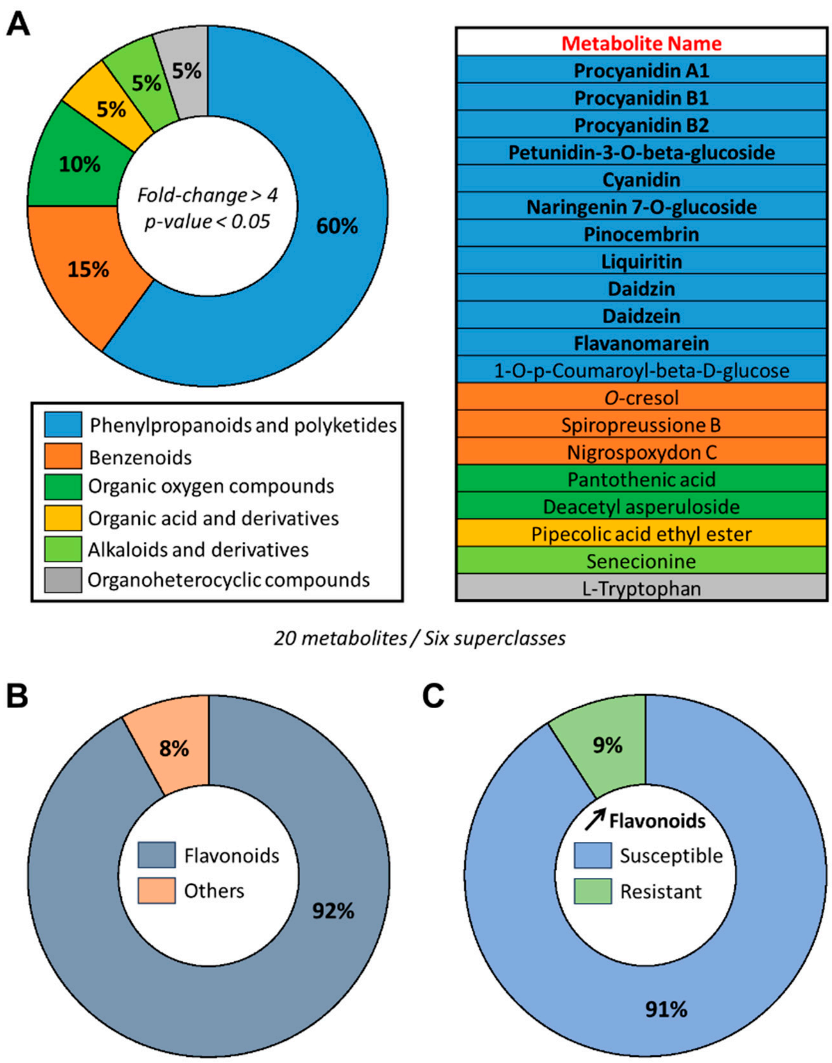

Figure S5. Classification and distribution of significantly altered metabolites among *Coffea* accessions. This figure included the 20 metabolites that satisfied the selection criteria of p-value < 0.05 and FC > 4. Metabolite classification into superclasses was performed using HMDB, PlantaeDB and KEGG. Since pipecolic acid ethyl ester and spiropreussione B are not listed in these three databases, their superclass assignments were inferred based on structural similarity to pipecolic acid and spiropreussione A, respectively. (A) Donut chart illustrating the distribution of 20 significantly altered metabolites across six chemical superclasses. Percentages represent the proportion of metabolites within each superclass relative to the total selected. Metabolites written in bold corresponds to flavonoids. (B) Donut chart indicating the proportion of flavonoids within the phenylpropanoids and polyketides superclass. (C) Donut chart depicting the percentage of flavonoids highly increased in the susceptible and resistant Coffea leaf accession.
